# COVID-19 pandemic: Revisiting the case for a dedicated financing mechanism for surgical care in resource-poor countries

**DOI:** 10.7189/jogh.11.03090

**Published:** 2021-07-31

**Authors:** Martilord Ifeanyichi, Jakub Gajewski, Rob Baltussen, Eric Borgstein, John Kachimba, Ruairi Brugha, Leon Bijlmakers

**Affiliations:** 1 Department for Health Evidence, Radboud Institute for Health Sciences, Radboud University Medical Centre, Nijmegen, the Netherlands; 2 EMAI Health Systems and Health Services Consulting, Nijmegen, the Netherlands; 3 Institute of Global Surgery, Royal College of Surgeons in Ireland, Dublin, Ireland; 4 College of Medicine, University of Malawi, Blantyre, Malawi; 5 Surgical Society of Zambia, Department of Surgery, University Teaching Hospital, Lusaka, Zambia; 6 Department of Public Health and Epidemiology, Royal College of Surgeons in Ireland, Dublin, Ireland

Due to the COVID-19 pandemic, health systems in most countries have struggled or buckled, as personnel, supplies, infrastructure and finances have been diverted to combat the outbreak, resulting in significant disruptions in delivery of health services [1]. International and national containment efforts interrupted the free flow of goods, restricted movements and imposed lock-downs, and prohibited all but essential services. Thereby, the pandemic triggered the worst economic recession since World War II [2], with the greatest impacts expected in resource-poor countries, given high pre-existing health and socioeconomic vulnerabilities.

Early projections suggested that low- and middle-income countries would experience 253 500 additional child deaths and 12 200 additional maternal deaths in six months [1]; a substantial proportion of these are related to disruptions in obstetric services. However, little attention has been given to the impact of COVID-19 on the broader safe surgical, obstetric, and anesthesia care (collectively termed ‘surgical care’) in resource-poor countries where 95% of the population lack access to such care [3]. Highlighting the exacerbating impact of the pandemic on access to surgery, this paper argues for a dedicated financing mechanism for surgical care, with a particular focus on district hospitals, which are the first points of comprehensive health care for rural populations.

In 2015, the Lancet Commission on Global Surgery (LCGS) published its report highlighting, among others, global inequities in access to surgery [4]. It proposed that resource-poor countries develop and implement National Surgical, Obstetric and Anaesthesia Plans (NSOAPs), as policy instruments within overall national health plans, for coordinating the resources and actors needed for essential surgical care scale-up. However, without evidence to guide design and implementation, and without adequate funding, such plans will remain just words on paper.

## HOW COVID-19 IMPACTS SURGERY

So why focus on surgery in resource-poor countries? First, there is the need. Cancellations of elective surgical procedures have lengthened waiting lists [5]; underlying conditions and patient quality of life have deteriorated; and the risks and likely volume of irreversible morbidity and premature deaths have increased [6]. Lockdowns have had direct effects, preventing people from accessing essential emergency surgery, as well as indirect effects, such as changes in the patterns of trauma. Increased incidences of domestic violence, impacting women in particular, could require surgical responses [7]. Where surgical care is available, the additional economic burden of the costs of personal protective equipment for hospital staff is often imposed on patients [8], in situations where most people were already facing financial catastrophes in accessing surgical care prior to the COVID-19 pandemic [9]. A modeling study estimated cancellations of 68%-73% of all elective surgeries globally just within 12-weeks of peak service disruption [10]; and that an estimated 20% increase in service delivery after the pandemic would clear the backlogs in 45 weeks [10]. Resource-poor countries have neither the resilience nor the resources to achieve this unaided.

**Figure:**
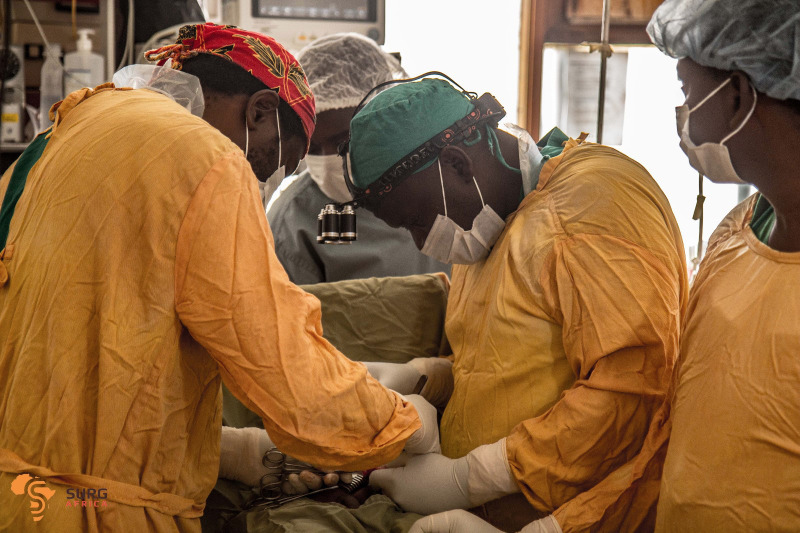
Photo: Scaling up Safe Surgery for District and Rural Populations in Africa (SURG-Africa). SURG-Africa was a 4-year implementation research project to scale up safe accessible surgery for district and rural populations in Tanzania, Malawi and Zambia (from the study group’s own collection, used with permission).

Second, there are the COVID-19 impacts on surgical responses. During the pandemic, improvisations and innovations including telemedicine have proliferated to mitigate clinical service disruptions [11]. Digital platforms are increasingly used for consultations, drug prescriptions and deliveries, management of chronic conditions, and patient referrals [11]. However, surgical operations by their nature must necessarily be undertaken in functioning hospital operating theaters, some of which have been repurposed to provide respiratory support to COVID-19 patients [6]. Surgery is complex, requiring a multidisciplinary team (including but not limited to surgeons, anesthesia providers and theater nurses), robust medical and non-medical supply chains, reliable equipment and instruments, an efficient information system, and sufficient financing. These constitute multiple stress points for service disruption during crises [12].

Third, failure to address the growing burden of surgical mortality and morbidity in resource-poor countries will have significant negative economic impacts in these countries [4]. Unlike many other non-communicable diseases, conditions such as trauma and obstetric emergencies, which are amenable to surgery, impact particularly adolescents and young adults; and thereby the economic productivity of families, communities and countries. Unless district surgical systems are restored and strengthened, the achievement of not only Sustainable Development Goal (SDG) 3 (good health and well-being), but also SDG 1 (ending poverty) will be undermined, further delaying the likelihood of achieving UHC and global aspirations of inclusive development and shared prosperity.

## GLOBAL AND NATIONAL ACTIONS FOR SURGICAL CARE IN RESOURCE-POOR COUNTRIES

Wealthy countries are grappling with huge economic challenges due to the COVID-19 pandemic. However, the greater threat and need in poorer countries make a compelling case for external support to scale-up surgical care. In SSA, for example, the pandemic will cost tens of billions of dollars in gross domestic product, cause a 3 to 5% rise in national budget deficits, and lead to an overall increase in public debts of more than $100 billion [13]. These are costs that SSA countries cannot afford, risking a worse impact on health and health care than the structural adjustment policies of the 1980s. A recent World Bank discussion paper, “From Double Shock to Double Recovery - Implications and Options for Health Financing in the Time of COVID-19” [2], projects major constraints on fiscal spaces for health due to falls in government and household health expenditures and declines in international development assistance (IDA) in poor countries.

Although earlier analyses indicated that only modest fiscal space expansions and IDA were needed for countries to scale up surgery [14], the additional COVID-19-related constraints on fiscal spaces for health mean that scale-up would now require substantial external resources. As the world emerges from the COVID-19 pandemic, though perhaps years later in SSA, donors should revisit earlier calls for a dedicated global financing platform for surgery, as suggested by Reddy et al., learning lessons from the likes of Global Fund to Fight AIDS, Tuberculosis and Malaria, GAVI, the Vaccine Alliance, and the Children’s Investment Fund Foundation [15]. A Global Surgery Fund as championed by the Global Surgery Foundation for instance [16] could finance initiatives for addressing access and quality of care, and the scale-up of tested approaches, drawing on recent evidence - see: https://www.surgafrica.eu/outputs2.html.

To avoid the risk of verticalization and its distortionary effects on country health systems, a common problem reported with earlier global health initiatives, such external funding should be aligned to the broader national health sector plans through NSOAPs, where available. The Fund should incentivise and support countries that have none to develop and then implement surgical plans. The LCGS blueprint for a systems approach to scale-up of safe, timely and affordable surgical care has enabled the national governments of Zambia, Tanzania, Nigeria, Rwanda, and Madagascar to develop national surgical plans, and is guiding numerous others in the development process. A major gap however has been the dearth of surgical systems research, particularly at district hospital level, to inform the implementation of country plans [17]. Implementation research is crucial to develop and evaluate contextualized strategies. Tested models in Zambia, Tanzania and Malawi, in two successive EU-funded research projects, show that global collaborations, with central roles for local actors such as professional associations, can support country governments to lead such national surgical scale-up initiatives [17].

National governments more than ever have a responsibility to prioritise surgery and implement the LCGS recommendations on the financing of surgery [4]. As a first step toward the development or redesign of NSOAPs, each country should conduct a thorough needs assessment and prioritisation exercise. The pandemic with its shockwaves on national health care systems provides a unique opportunity for each country to systematically and critically evaluate surgical systems at specific stress points, perhaps using as a framework the health systems building blocks [12]. This could yield valuable evidence for scale-up and resource allocation decisions. Governments should make explicit budgetary allocations to surgical services at national, specialist referral hospital and district hospital levels, coupled with mechanisms for tracking expenditures. This will facilitate progress monitoring and foster accountability and transparency [4]. Countries such as Tanzania and Zambia have NSOAPs that propose the creation of budget lines for surgical care at district levels. These now need to be funded. Even though surgeries have been proven to be cost-effective, our experience from ten years of surgical systems research at district hospitals in SSA indicates that the current district health funding systems and levels provide disincentives for hospital managers to prioritise such resource-intensive services as surgeries [18]. Some form of performance-linked financing mechanism for surgical care at this level would be crucial to incentivize local providers to do more surgeries as and when the need arises.

## CONCLUSION

The COVID-19 pandemic has exacerbated the long neglect of surgical services in resource-poor countries, especially at the district level; and it has compounded the threat posed by diseases amenable to surgery to the long-term health, well-being and development of entire nations. The present crisis presents both a justification and an opportunity for a dedicated global funding mechanism for strengthening surgical capacities in resource-poor countries. We hope that these ideas will contribute to a dialogue among practitioners, policy makers, researchers, political leaders, as well as representatives of bilateral and global health organisations, funding agencies and non-governmental organisations, with a view to securing and committing resources to expedite universal access to surgical care.
